# Enhanced sensitivity of Au@Bi_2_WO_6_ flower-like materials to formaldehyde

**DOI:** 10.1186/s11671-023-03923-4

**Published:** 2023-11-13

**Authors:** Ruifeng Zhang, Lei Liu, Weiye Yang, Yao Liu, Yingkai Liu

**Affiliations:** 1 Yunnan Key Laboratory of Optoelectronic Information Technology, Kunming, 650500 People’s Republic of China; 2 Institute of Physics and Electronic Information, Yunnan Normal University, Kunming, 650500 People’s Republic of China; 3 Key Laboratory of Advanced Technique and Preparation for Renewable Energy Materials, Ministry of Education, Yunnan Normal University, Kunming, 650500 China

**Keywords:** Au@Bi_2_WO_6_ flower-like structure, Formaldehyde detection, Gas sensor, Sensitivity

## Abstract

Bi_2_WO_6_ flower-like materials (FMs) were prepared by a hydrothermal method, followed by an in-situ reduction method to prepare Au@Bi_2_WO_6_ FMs. X-ray diffraction, scanning electron microscopy, transmission electron microscopy, high-resolution transmission electron microscopy, and X-ray photoelectron spectroscopy were employed to characterize the samples. It was discovered that the calculated O_V_ content of Au@Bi_2_WO_6_ FMs is 25.16% whereas that of Bi_2_WO_6_ FMs is 20.81%, offering appropriate active sites for the absorption of gases and thus enhancing outstanding sensing property. Moreover, the detection of volatile and hazardous substances such as formaldehyde, methanol, acetone, benzene, toluene, and xylene was carried out to assess the efficacy of the Au@Bi_2_WO_6_ FMs sensors. The optimal operating temperatures for the Bi_2_WO_6_ FMs and Au@Bi_2_WO_6_ FMs sensors were 290 and 260 °C, respectively. Compared with Au@Bi_2_WO_6_ FMs sensor and Bi_2_WO_6_ FMs one, the best response of the front was 250 (900)–100 (800) ppm formaldehyde whereas that of the latter was 90 (230). Therefore, Au@ Bi_2_WO_6_ FMs have good response and selectivity, which are promising candidates for formaldehyde detection.

## Introduction

People are becoming more and more worried about environmental protection and health as society develops. Various volatile organic compounds (VOCs) are emitted in industries, which destroy the environment and harm human health [1, 2]. Formaldehyde (HCHO) gas has received lots of attention [3] in that it is one of the more prevalent hazardous VOCs in dwellings, workplaces, and public spaces. It is easy to arouse malignant disorders such as cancer and leukemia [4] because of long-term exposure to HCHO gases. In recent years, increasing VOCs used in construction and remodeling has lead to the delayed release of HCHO to the environment [5]. Therefore, it is required to reliably detect HCHO gas for human health in new dwellings, and places of work.

Metal oxide semiconductor (MOS) sensors exhibited tremendous promise for identifying various gases due to their inexpensiveness, easy operation, and mobility [6]. Many MOS sensors including ZnO [7], In_2_O_3_ [8], SnO_2_ [9], Co_3_O_4_ [10], CuO [11], and TiO_2_ [12] are utilized to monitor VOCs. However, multi-metallic oxide sensors are generally recognized to be more durable and dependent in comparison to binary metal oxide sensors. At present, Bi_2_WO_6_ is successfully used as sensor material owing to its exceptional electron transmission efficacy, crystal structure stability, and compatibility with the environment [13–15]. Yuan et al. [16] prepared assemble hierarchical Bi_2_WO_6_ by the surfactant-assisted hydrothermal method, demonstrating excellent gas sensing characteristics toward ethanol. Cao et al. [17] applied a one-step hydrothermal technique to create a new dandelion-like Bi_2_WO_6_ and investigated its response to the lowest detection limit of ethylene glycol with 1 ppm at 270 °C. Yun et al. [18] produced a triethylamine sensor based on bulk Bi_2_WO_6_ that has an ultrafast response-recovery speed at 240 °C and large response of 30.19–100 ppm triethylamine gas. Despite numerous literatures on Bi_2_WO_6_ sensing materials, pure Bi_2_WO_6_ materials still have elevated operating temperatures as well as less fortunate VOC selectivity, implying that the structure and detection properties of pure Bi_2_WO_6_ need further enhanced and optimized.

For the past few years, there has been significant research on the development of sensors utilizing composite nanomaterials consisting of noble metals and semiconductors. The reason is that noble metal nanoparticles (NPs) enhance the adsorption capacity of the sensing material on the target gas molecules, which in turn leads to change in resistance. As illustrated by Liu et al. [19], Au@SnO_2_ sensors are 3 times more responsive than pure SnO_2_ sensors and have faster response and recovery times. It is reported that the ZnO nanostructures coated with 6% Au NPs sensor possessed 9 times response to 100 ppm acetone vapor at 280 °C than pristine ZnO [20]. Li et al. [21] documented that the Au@LaFeO_3_ detector displayed 27 times larger response to 100 ppm ethanol than the pure LaFeO_3_ detector at the best working temperature. Cheng et al. [22] also revealed that the Au@CuO FMs sensor possesses 7 times higher response than the CuO FMs sensor to 1000 ppm ethanol. As a result, it concluded that Au NPs can effectively improve the response and recovery characteristics of gas sensors. To the best of our knowledge, no one has investigated the sensor based on Au NPs adorned Bi_2_WO_6_ FMs.

In this work, we have developed a sensor based on the Au@Bi_2_WO_6_ FMs by attaching Au nanoparticles to the surface of Bi_2_WO_6_ FMs. It demonstrated that this sensor has an ability to detect organic substances like formaldehyde, methanol, acetone, benzene, toluene, and xylene. It is found that the optimal working temperature decreases from 290 to 260 °C and the sensor had a fourfold larger response to 800 ppm formaldehyde than the untreated one. Additionally, it has a strong reactivity towards several gases including methanol, acetone, benzene, toluene, and xylene. However, the enhanced effect has a lesser magnitude than formaldehyde. The Au@Bi_2_WO_6_ FMs sensor exhibited improved sensitivity and selectivity towards HCHO vapor at its optimal temperature.

## Experimental section

### Chemicals and materials

Bi(NO)_3_, Na_2_WO_4_·2H_2_O, HAuCl_4_·4H_2_O, Methanol, benzene, toluene, xylene, acetone, and formaldehyde had been bought from Tianjin Sailboat Chemical Reagent Technology Co., Ltd. (Tianjin, China). All materials and reagents are analytical grades and used without any treatment.

### The synthesis of Bi_2_WO_6_ and Au@ Bi_2_WO_6_ FMs

Synthesis of Bi_2_WO_6_ FMs was as follows: firstly, 1.101 g of Bi(NO_3_)_3_ was dissolved in 30 mL of deionized water and stirred for 25 min to obtain solution A. In another vessel, 0.367 g of Na_2_WO_4_·2H_2_O was added to 45 mL of DI water and stirred for 25 min to obtain solution B. Solution B was added to solution A and stirred for 55 min at 40 °C to get solution C. Secondly, solution C had been moved to a 100 mL Teflon-lined stainless steel autoclave while kept for 16 h at 160 °C. After that, let it naturally cool to room temperature. The resulting solution was separated by centrifugal and rinsed several times using DI water and ethanol. Finally, the materials were dried in an oven at 60 °C for 12 h to attain the white powder.

To prepare Au@Bi_2_WO_6_ FMs, a suitable amount of Bi_2_WO_6_ FMs powder was dispersed in DI water and followed by 2 mL of PVP water-soluble solution and 6 mL of HAuCl_4_·4H_2_O solution. Both of solutions were completely mixed for 3 min and then injected by 8 mL of ascorbic acid solution. As a result, the solution quickly turned purple and was agitated for 3 h. The entire mixture was centrifuged and then rinsed and dried to yield Au@Bi_2_WO_6_ FMs.

### Construction of the sensors

First, the surface of the Au-forked finger electrode was cleaned with acetone, ethanol, and DI water. Subsequently, a mixture of 0.05 g sample and 1 mL deionized water was prepared. We extracted 30 µL paste from the mixture and then uniformly applied it onto the gold electrode substrate by a paint pen. Thirdly, the treated electrode sheets were aged for 12 h at 290 °C for Bi_2_WO_6_ FMs and at 260 °C for Au@ Bi_2_WO_6_ FMs. The distance between the electrodes was around 50 µm. The surface area of the sensor was approximately 10 mm × 10 mm. We fabricate a series of 20 Bi_2_WO_6_ FMs and Au@Bi_2_WO_6_ FMs gas sensors to test each of the device's performance parameters. The results show that the repeatability of each performance indicator is more than 80%.

### Measurement of sensors

The evaluation of the sensor’s performance is conducted by the CGS-1TP Intelligent Gas Sensing and Analysis System (manufactured by Beijing Elite Tech Co., Ltd.) at the specified concentration of VOCs. The CGS-1TP system comprises three essential components: the gas chamber, the platform and the main unit. The sensor was put on the temperature control platform, which was connected to a probe station. The gaseous VOCs environment is established through the introduction of VOCs liquid (with its volume calculated through the solution dispensing applications known as EtLiquidVolume) to a liquid vaporizer. The test software known as the CGS-1TP Highly intelligent gas-sensitive inspection arrangement, is responsible for collecting the measured data. The interdigitated electrode coating of the sample is in intimate contact with the temperature stage. After the temperature of the temperature control platform rises to the set value and the resistance of the sensor is stable, inject the calculated liquid into the air chamber (18 L). After the resistance is stable, open the chamber to restore the resistance of the sensor to the original level. All the tests are conducted in an environment with a temperature of 25 °C and a humidity of 38–39% RH. The temperature of the sample stage is controlled at 220–320 °C. The rest of the evaluation of the selectivity, stability and response recovery time of the gas sensors are carried out at the optimal operating temperature.

## Results and discussions

### Characterization

**Fig. 1:**
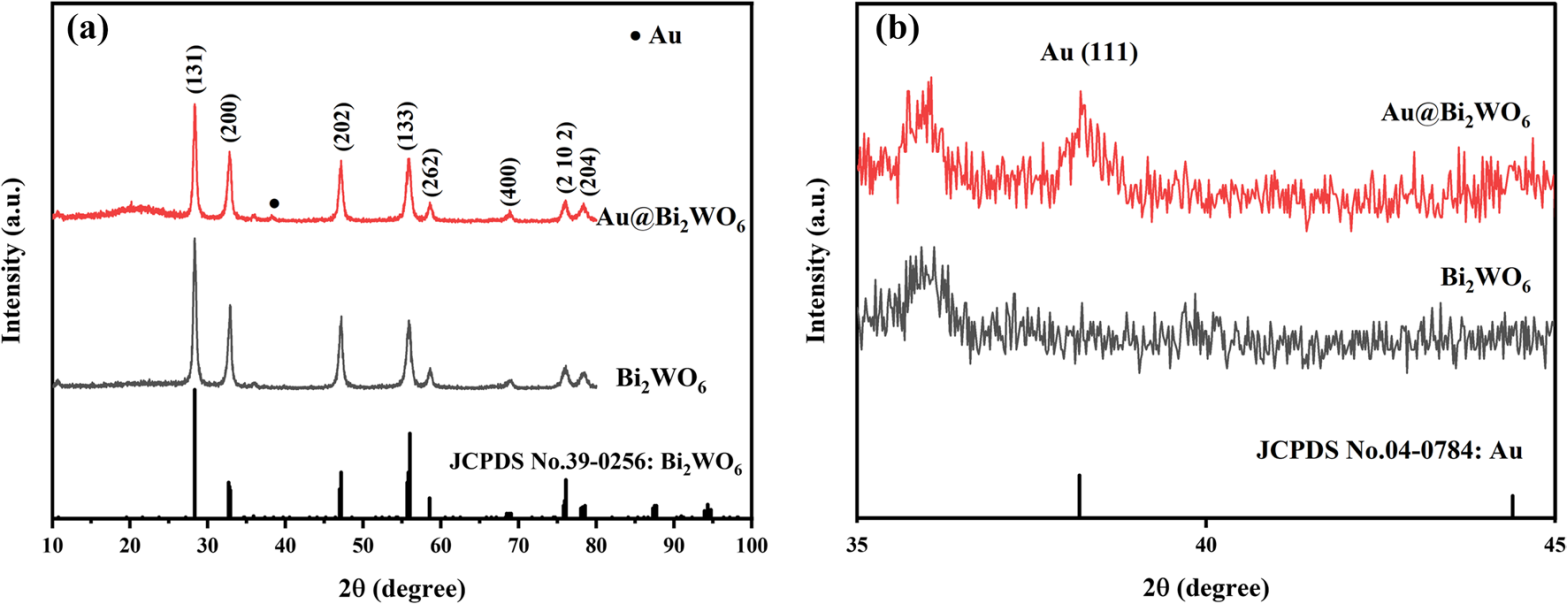
**a** Bi_2_WO_6_ and Au@Bi_2_WO_6_ FMs XRD patterns and **b** their local amplification spectrum

The XRD patterns of Bi_2_WO_6_ FMs and Au@Bi_2_WO_6_ FMs are shown in Fig. 1a. The diffraction peaks located at 2θ = 28.2°, 32.7°, 47.1°, 55.9°, 58.5°, 68.7°, 76°, and 78.5°correspond to the (131), (200), (202), (133), (262), (400), (2 10 2), and (204) crystal planes respectively (JCPDS 39-0256) [23]. The diffraction peak of 2θ = 38.1° corresponds to the Au crystal plane (111) (JCPDS 04-0784), as shown in Fig. 1b. No other peaks were detected, indicating the Au NPs were effectively decorated on the Bi_2_WO_6_ FMs. The well-defined diffraction peaks of Au@ Bi_2_WO_6_ FMs revealed excellent crystallinity. The average grain size of Au NPs can be calculated by Scherrer's equation d = $\frac{k\lambda }{\beta \mathit{cos}\theta }$, where λ, β, and θ are X-ray wavelength, the half-maximum width of the spread XRD lines and diffraction angle, $k$ is a constant. The average diameter of Au NPs is estimated to be 15.3 nm.

**Fig. 2:**
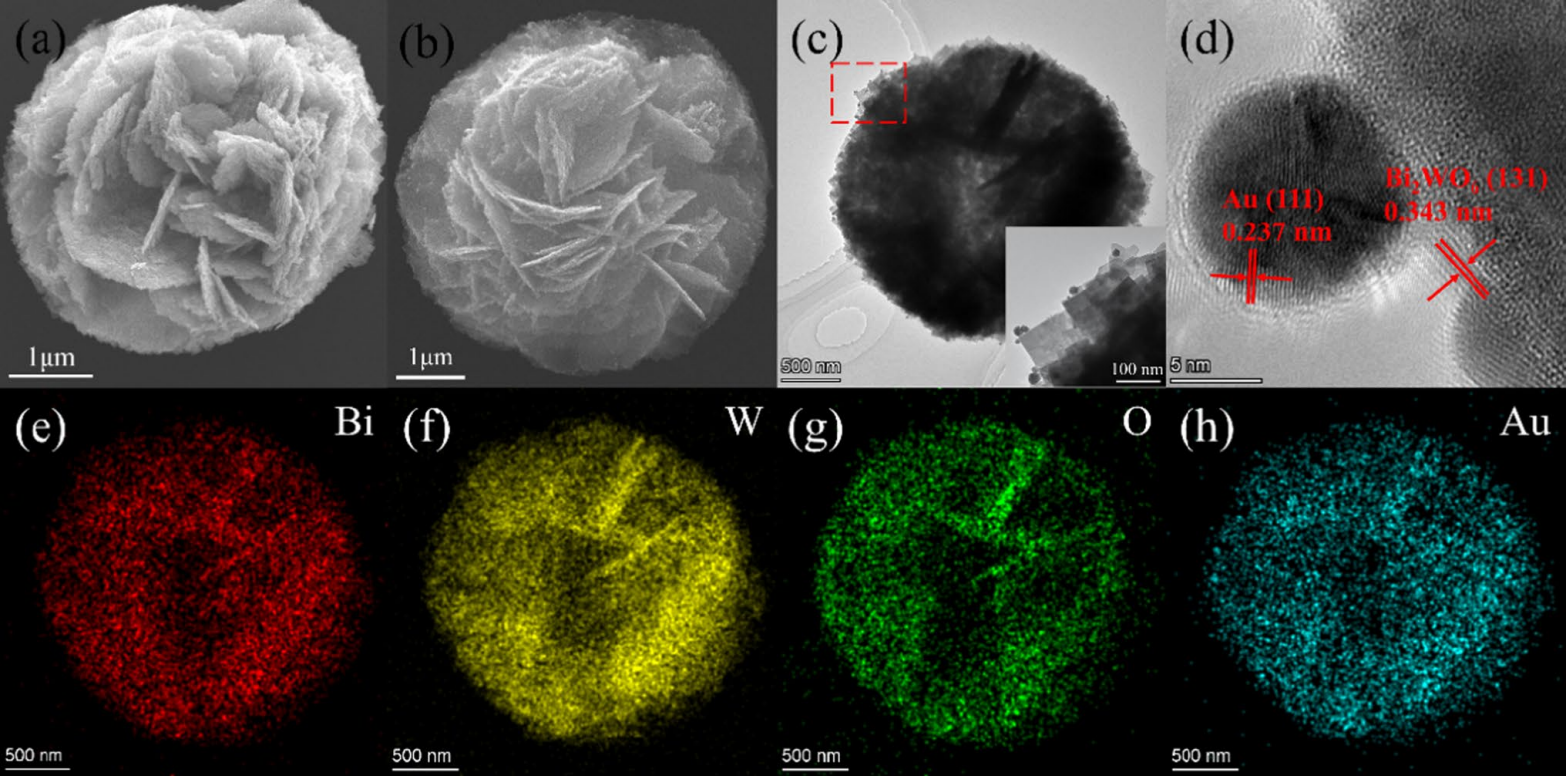
SEM, TEM, HRTEM and EDX mapping images of the samples. **a** SEM of Bi_2_WO_6_ FMs; **b**–**h** SEM, TEM, HRTEM and EDX mapping of Au@Bi_2_WO_6_ FMs

Figure 2a displays SEM image of Bi_2_WO_6_ FMs. It is seen that it consists of many square-shaped building blocks. In Fig. 2b, Bi_2_WO_6_ FMs were reduced to Au@Bi_2_WO_6_ FMs by an in-situ reduction reaction of HAuCl_4_·4H_2_O solution. Its TEM image was illustrated in Fig. 2c. It noted that Au NPs were clearly observed, revealing that Au NPs with diameters of between 5–25 nm are firmly attached to the surface of Bi_2_WO_6_ FMs. Its HRTEM images are shown in Fig. 2d. The lattice spacing of Bi_2_WO_6_ FMs is discovered to be 0.343 nm, which corresponds to the (131) crystal plane, whereas the lattice spacing is 0.237 nm, matching with the (111) crystal plane of Au. Energy dispersive X-ray (EDX) mappings of it were depicted in Fig. 2e–h. It demonstrated that Bi, W, O, and Au elements uniformly distributed throughout the Au@Bi_2_WO_6_ FMs.

**Fig. 3:**
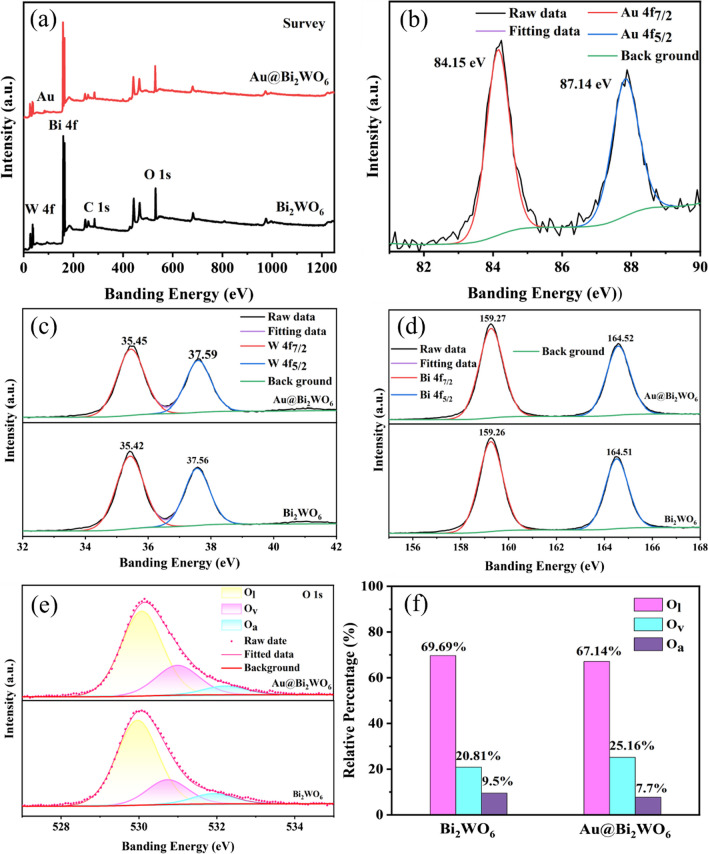
The XPS spectra of the Bi_2_WO_6_ and Au@Bi_2_WO_6_ FMs. **a** A survey spectrum; **b** Au 4 f spectra; **c** Bi 4 f spectra; **d** W 4 f spectra; **e** O 1 s spectra; **f** fit results of O 1 s XPS spectra of Bi_2_WO_6_ and Au@Bi_2_WO_6_

Figure 3 depicts XPS spectra of Bi_2_WO_6_ FMs and Au@Bi_2_WO_6_ FMs. It presents that the sample contains Au, Bi, W, and O elements, as shown in Fig. 3a. Figure 3b shows two binding energy peaks designated as Au 4f_7/2_ and Au 4f_5/2_, respectively [22], suggesting the presence of Au in the sample. Figure 3c describes Bi4f XPS spectra of Bi_2_WO_6_ FMs and Au@Bi_2_WO_6_ FMs. The binding energies of Bi 4f_7/2_ and Bi 4f_5/2_ are 159.26 and 164.51 eV for Bi_2_WO_6_ FMs, whereas those of Bi 4f_7/2_ and Bi 4f_5/2_ are 159.27 and 164.52 eV for Au@Bi_2_WO_6_ FMs. The binding energies are related to the Bi^3+^ oxidation state [24]. The W4f XPS spectra of Bi_2_WO_6_ FMs and Au@Bi_2_WO_6_ FMs are denoted in Fig. 3d. The binding energies of the W4f_7/2_ and W4f_5/2_ are 35.42 and 37.56 eV for Bi_2_WO_6_ FMs. Those of the W4f_7/2_ and W4f_5/2_ are 35.59 and 37.59 eV for Au@ Bi_2_WO_6_ FMs, which corresponds the oxidation state of W^6+^ [24]. The O 1 s spectra of Bi_2_WO_6_ FMs and Au@Bi_2_WO_6_ FMs are presented in Fig. 3e. Three distinct peaks were detected, corresponding to oxygen lattice (O_l_), oxygen vacancy (O_V_), and dioxygen adsorbed (O_a_). The relative proportions of each type of oxygen in Bi_2_WO_6_ FMs and Au@Bi_2_WO_6_ FMs are displayed in Fig. 3f. Notably, the calculated content of O_V_ in Au@Bi_2_WO_6_ FMs (25.16 at.%) surpasses that of Bi_2_WO_6_ FMs (20.81 at.%). The presence of O_V_ is known to be crucial in gas sensitive reactions as it facilitates gas adsorption and enhances sensing capabilities. Consequently, it is plausible to expect that Au@Bi_2_WO_6_ FMs exhibit better gas sensing execution as compared to Bi_2_WO_6_ FMs.

**Fig. 4:**
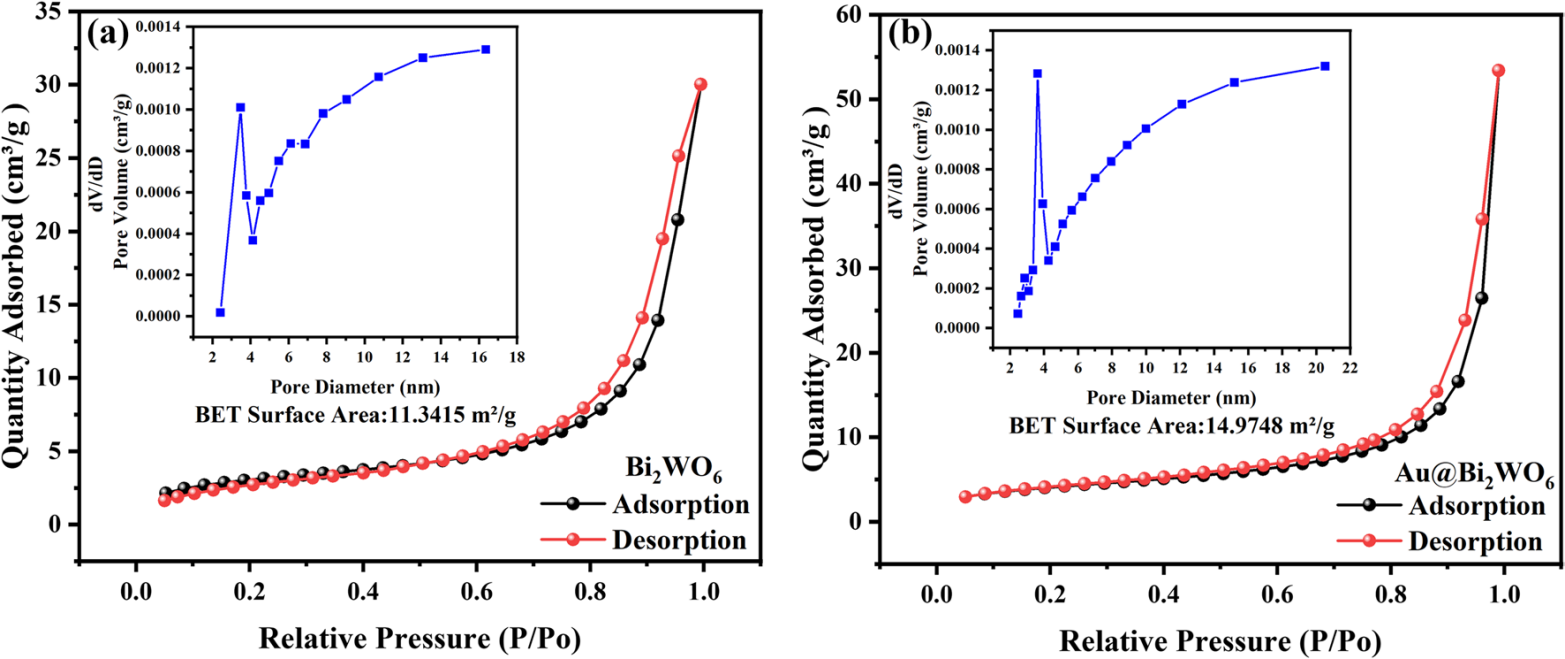
The N_2_ adsorption–desorption isotherms and their BJH pore size distributions of the samples. **a** Bi_2_WO_6_ FMs; **b** Au@Bi_2_WO_6_ FMs

Figure 4 presents the N_2_ adsorption–desorption isotherms of Bi_2_WO_6_ FMs and Au@Bi_2_WO_6_ FMs, aiming to elucidate the impact of Au NPs on the specific surface area and BJH pore size distribution of Bi_2_WO_6_ FMs. Based on the categorization established by the IUPAC, the elucidated isotherms of the samples were determined to be of type IV, thereby confirming the existence of mesopores. The specific surface area and pore size were 11.3415 m^2^/g and 11.2596 nm (Bi_2_WO_6_ FMs) and 14.9748 m^2^/g and 8.9365 nm (Au@Bi_2_WO_6_ FMs), respectively, indicating a significant effect of Au complexation on the pore size of Bi_2_WO_6_ FMs. The Au@Bi_2_WO_6_ FMs has a large specific surface area and a relatively small aperture range. The result may be due to the hollow and porous structure of the materials. The specific surface area of the composite sample increases, which is very beneficial to the adsorption of the target gas [25, 26]. Consequently, it is reasonable that the Au@Bi_2_WO_6_ FMs sensor might potentially provide superior gas performance.

### Gas sensing property

#### Gas sensing performance of Bi_2_WO_6_ FMs and Au@Bi_2_WO_6_ FMs sensors

**Fig. 5:**
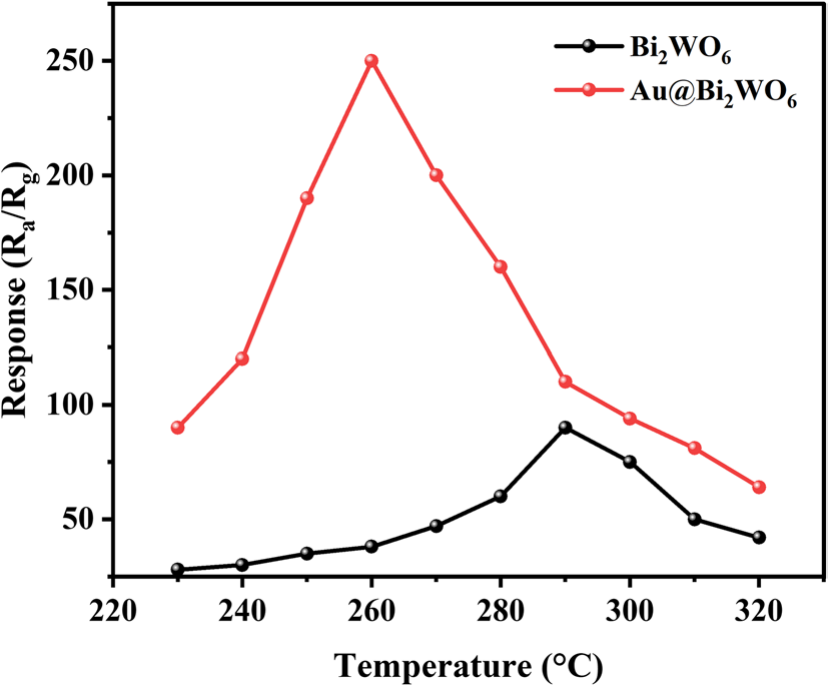
The response (R_g_) versus temperature (T) of Bi_2_WO_6_ FMs and Au@Bi_2_WO_6_ FMs to formaldehyde at 100 ppm

Figure 5 depicts the response against temperature (T) curves of the Bi_2_WO_6_ FMs and Au@Bi_2_WO_6_ FMs sensors to 100 ppm formaldehyde. S = R_a_/R_g_, where R_a_ is the resistance of the target gas and R_g_ is the resistance of the sensor in the target gas [27], can be used to calculate the response. When the Bi_2_WO_6_ FMs are modified with Au NPs, the ideal working temperature drops from 290 to 260 °C. The device's low operating temperature contributes to its long lifetime and low energy consumption.

**Fig. 6:**
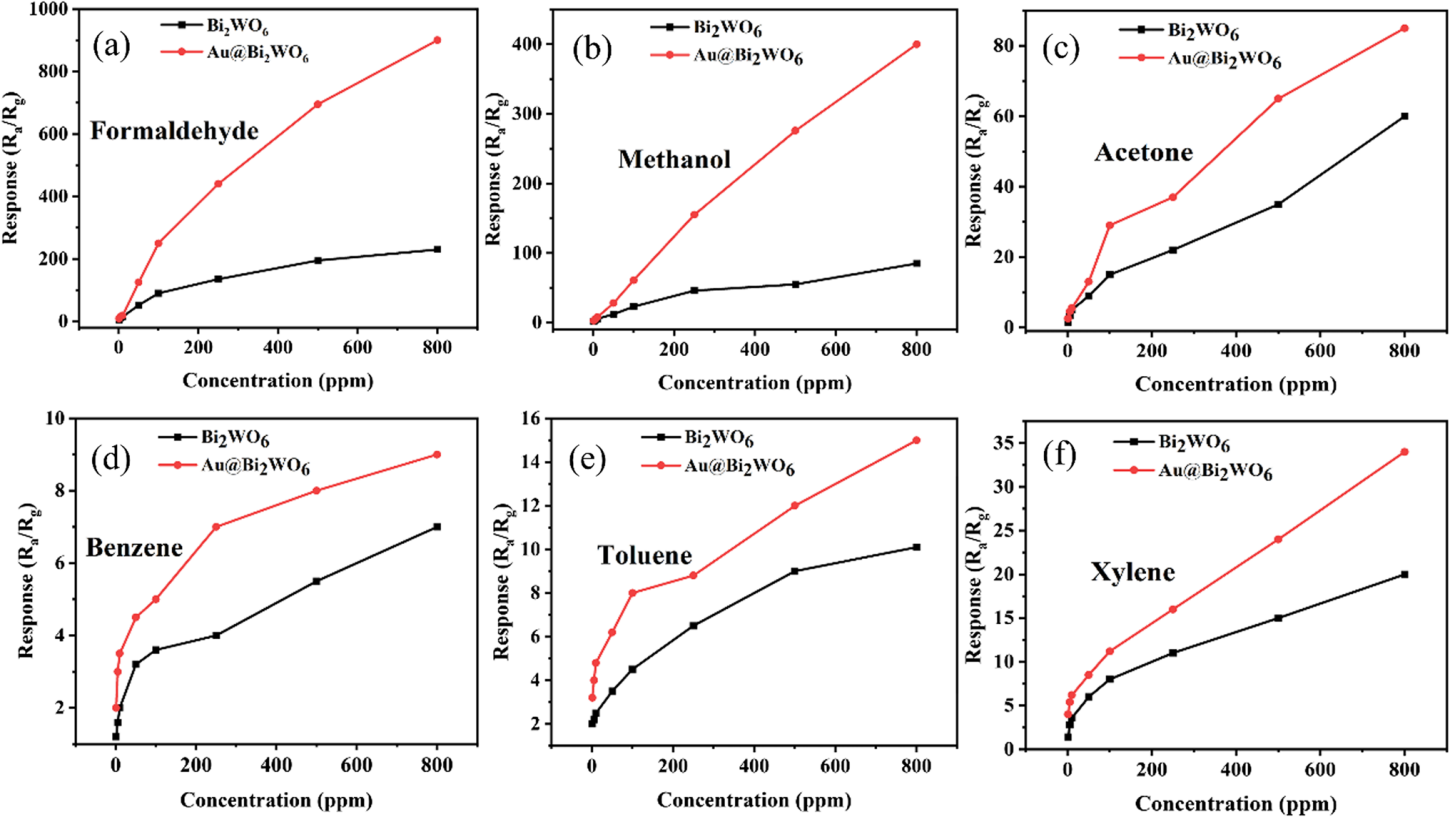
Response of Bi_2_WO_6_ FMs and Au@Bi_2_WO_6_ FMs sensors to different gas concentrations. **a** Formaldehyde; **b** methanol; **c** acetone; **d** benzene; **e** toluene, and **f** xylene

**Fig. 7:**
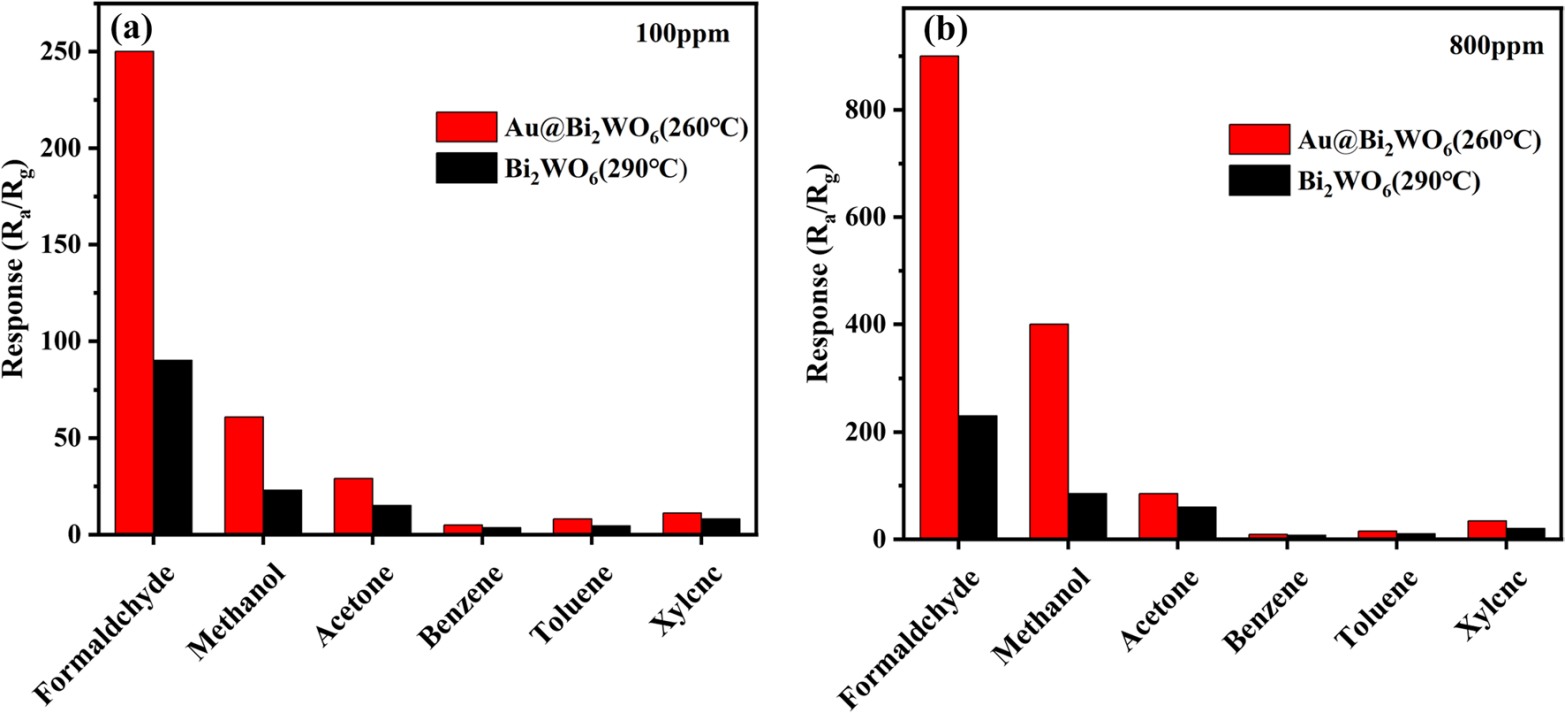
Sensing performance of Bi_2_WO_6_ FMs and Au@ Bi_2_WO_6_ FMs sensor for 6 VOCs gas at **a** 100 ppm and **b** 800 ppm

The sensing properties of Bi_2_WO_6_ FMs sensors with and without Au NPs alteration to formaldehyde, methanol, acetone, benzene, toluene, and xylene were examined. Figure 6 envisages the reactions of the Bi_2_WO_6_ FMs and Au@Bi_2_WO_6_ FMs sensors to various vapor concentration at 290 and 260 °C, respectively. Their response to six different VOCs is found to increase with increasing vapor concentrations. The responses of the Bi_2_WO_6_ FMs sensor were only 90, 23, 15, 3.6, 4.5, and 8–100 ppm formaldehyde, methanol, acetone, benzene, toluene, and xylene, respectively whereas those of the Au@Bi_2_WO_6_ FMs sensor reached 250, 61, 29, 5, 8 and 11.2, respectively, as shown in Fig. 7a. The Au@Bi_2_WO_6_ FMs sensor exhibited outstanding sensing performance. To our surprise, their response was up to 250/900 to 100/800 ppm formaldehyde. The details are that the responses of the Bi_2_WO_6_ FMs sensor were only 230, 85, 60, 7, 10.1, and 20 to 800 ppm of formaldehyde, methanol, acetone, benzene, toluene, and xylene, respectively. However, those of the Au@Bi_2_WO_6_ FMs sensor increased to 900, 400, 85, 9, 15 and 34, as depicted in Fig. 7b. Thus, the Au@Bi_2_WO_6_ FMs sensor is exceedingly sensitive to formaldehyde at low and high concentration and its response to formaldehyde is much greater than other gases.

**Fig. 8:**
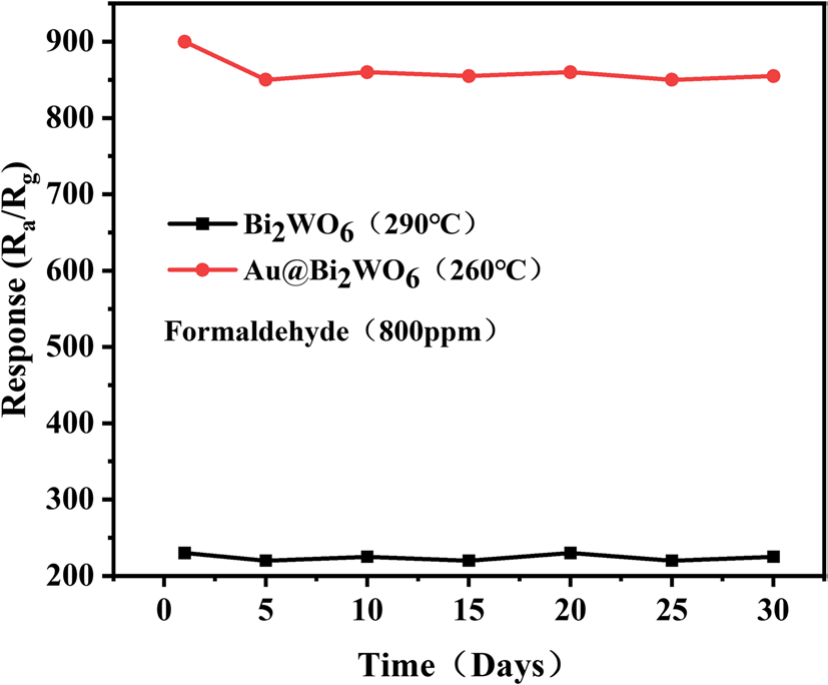
Repeatability and stability of the sensor

To assess the repeatability and dependability of the sensors, an investigation was conducted on two sensors to 800 ppm of formaldehyde over 30 days. The result is presented in Fig. 8. It told us that the two sensors exhibited a notable level of stability and a high degree of reproducibility.

**Fig. 9:**
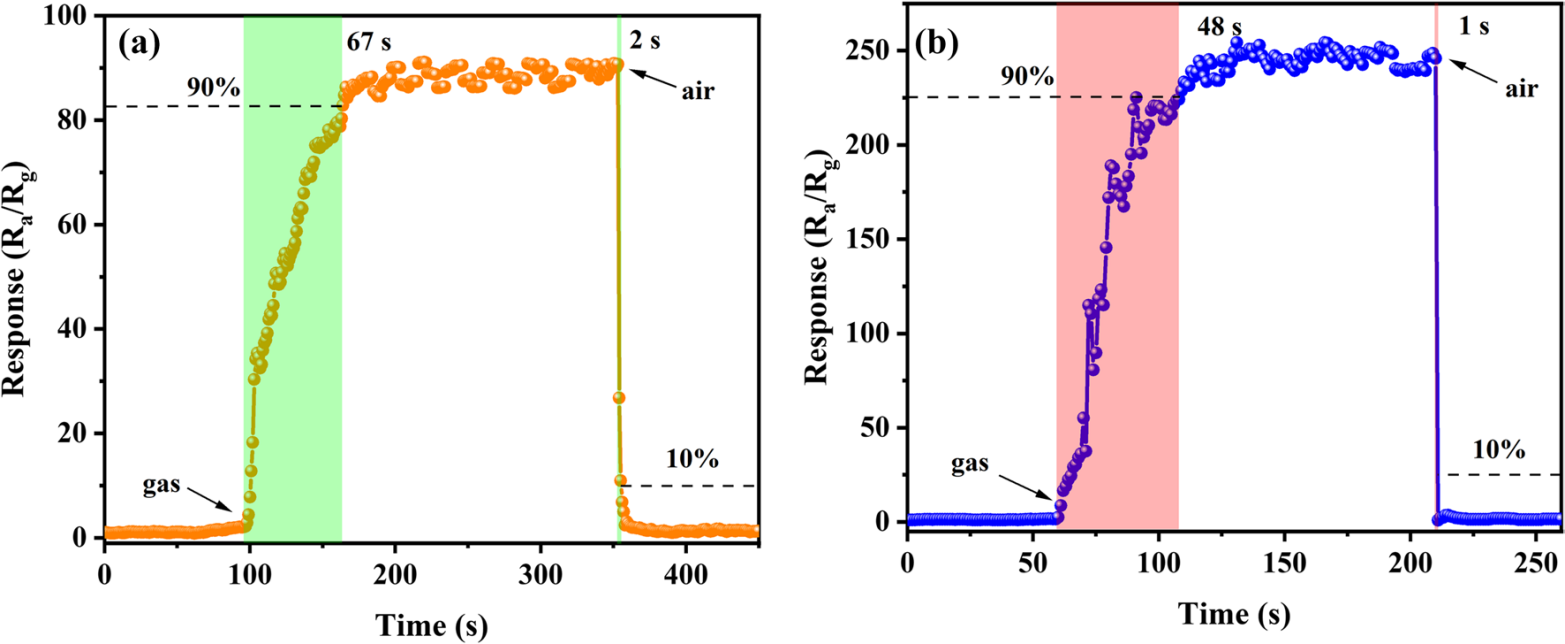
Response and recovery times of **a** Bi_2_WO_6_ FMs and **b** Au@Bi_2_WO_6_ FMs for 100 ppm formaldehyde at its optimal operating temperatures

Response/recovery time is another critical figure of merit for sensor. To study the sensitive dynamics of it, we have investigated the response/recovery time of Bi_2_WO_6_ FMs and Au@Bi_2_WO_6_ FMs sensors for 100 ppm formaldehyde at their optimal operating temperatures. It can be defined as the time taken for the response to increase from 10 to 90% of the peak value and versa, respectively. The response time of the Au@Bi_2_WO_6_ FMs sensor at 100 ppm formaldehyde was reduced from 67 to 48 s, and the recovery time was reduced from 2 to 1 s, as displayed in Fig. 9. The Au@Bi_2_WO_6_ FMs sensor has a faster response/recovery time duo to the presence of Au NPs.

Table 1 shows comparison of Au@Bi_2_WO_6_ FMs with previously reported sensors. It was discovered that our Au@Bi_2_WO_6_ FMs sensor outperformed the reported sensors. As a result, the prepared Au@Bi_2_WO_6_ FMs have a high potential for gas detection in real-world applications.

**Table 1:** Gas sensitive properties of different sensor materials for formaldehyde

Sensing materials	Temperature (℃)	Concentration (ppm)	Response	References
Sn_0.959_La_0.055_O_2_ nanospheres	200	100	149.59	[28]
SnO_2_ Nanostructures	275	100	53.6	[29]
3D porous SnO_2_	230	50	27	[30]
CdGa_2_O_4_ nanospheres	140	100	99	[31]
CdO–ZnO nanorices	350	300	34.5	[32]
Pt/CuBi_2_O_4_ nanospheres	180	5	8	[33]
Zn_2_SnO_4_/ZnO composites	160	100	22.5	[34]
In_2_O_3_/Co_3_O_4_ core/shell nanofibers	180	100	15.7	[35]
Ca-doped ZnO nanoparticles	250	5	5.28	[36]
Au/LaFeO_3_ nanocomposites	200	10	16.1	[37]
PdO-ZnO/SnO_2_ nanoparticles	140	10	5.3	[38]
Bi_2_WO_6_ FMs	290	100	90	This work
Au@Bi_2_WO_6_ FMs	260	100	250	This work

#### Theoretical limit of detection (LOD)

**Fig. 10:**
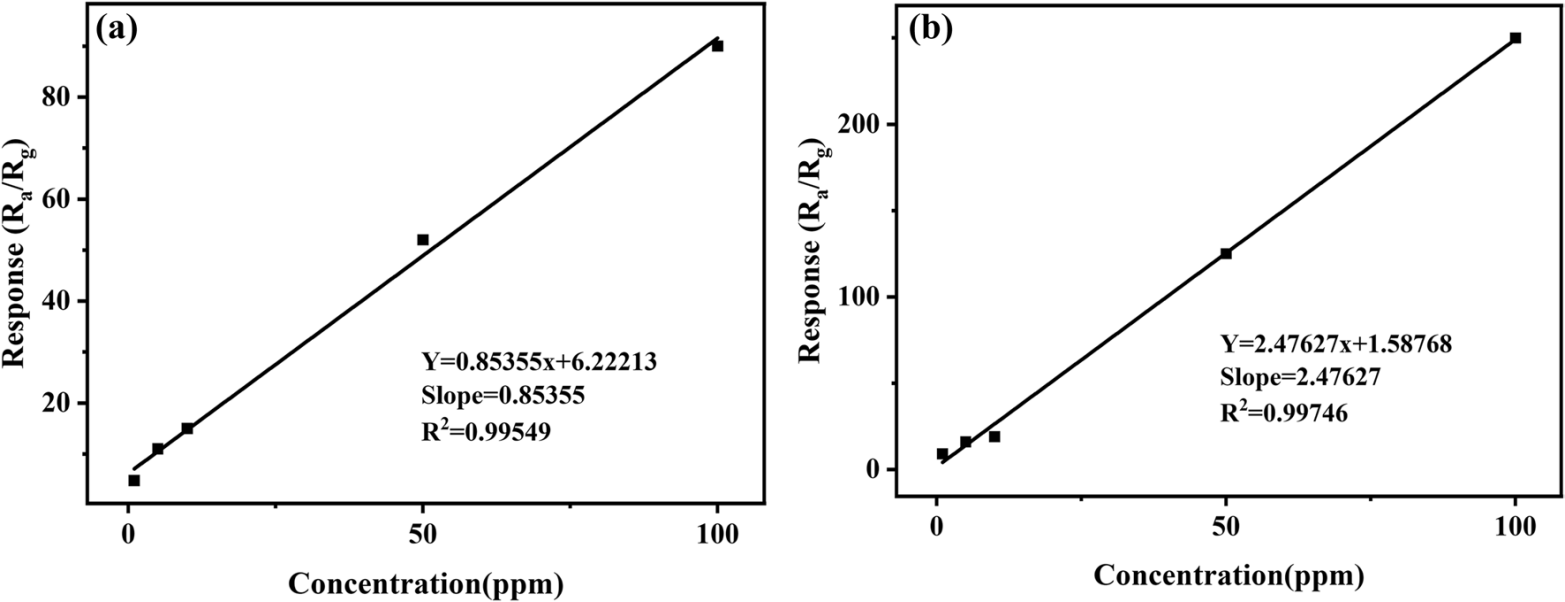
The linear fitting line for formaldehyde concentration and response in the range of 1–100 ppm. **a** Bi_2_WO_6_ FMs gas sensor; **b** Au@ Bi_2_WO_6_ FMs gas sensor

Figure 10 highlights a linear fitting of the response of formaldehyde gas with a concentration of 1–100 ppm. In the selected gas concentration range, the response has a good linear relationship with the gas concentration with R^2^ = 0.99549 and the slope of 0.85355 ppm^−1^. Sixty points (N = 60) are randomly selected from the baseline of the original data, and the square of the standard deviation is calculated as S^2^ = 0.04846. The formula RMS_noise_ =$\sqrt{\frac{{S}^{2}}{N}}$ is used to obtain RMS_noise_ = 0.02842 of the sensors to HCHO and the theoretical limit of detection (LOD) is 99.9 ppb (LOD = 3 × $\frac{RM{S}_{noise}}{Slope}$) for the Bi_2_WO_6_ FMs sensor. In a comparable vein in Fig. 10b, R^2^ for the Au@Bi_2_WO_6_ FMs sensor is 0.99746. Its LOD is 20.4 ppb. Au NPs greatly reduce the LOD of Au@Bi_2_WO_6_ FMs sensor to HCHO. The response value of the Au@Bi_2_WO_6_ FMs gas sensor for 80 ppb formaldehyde gas is 1.42, which is consistent with the theoretically calculated detection limit.

### Sensing mechanism

A possible mechanism is related to the principle of surface control, which is benefit to gas adsorption, charge transfer, and desorption [39]. When the gas sensor is in the air, atmospheric oxygen is absorbed by the sample and adheres to the surface of the sample, keeping it in a steady state equilibrium. As the sample temperature continues to rise, the following ionization reaction will occur [40].

1 $${\text{O}}_{{\text{2(gas)}}} \to {\text{O}}_{{\text{2(ads)}}}$$

2 $${\text{O}}_{{\text{2(ads)}}} {\text{ + e}}^{ - } \to {\text{O}}_{{\text{2(ads)}}}^{ - } \;\left( {{\text{T}} < {1}00\,^\circ {\text{C}}} \right)$$

3 $${\text{O}}_{{\text{2(ads)}}}^{ - } {\text{ + e}}^{{}} \to {\text{2O}}_{{\text{(ads)}}}^{ - } \;\left( {{1}00\,^\circ {\text{C}} < {\text{T}} < {3}00\,^\circ {\text{C}}} \right)$$

4 $${\text{O}}_{{\text{(ads)}}}^{ - } {\text{ + e}}^{ - } \to {\text{O}}_{{\text{(ads)}}}^{{{2} - }} \,\left( {{\text{T}} > {3}00\,^\circ {\text{C}}} \right)$$

Oxygen adsorbs on the surface of the sample, which will interact with the formaldehyde gas and produce the release of the trapped electrons, resulting in lower resistance [41, 42] when the gas sensor is exposed to formaldehyde vapor. In the process of target gas interaction, formaldehyde molecules react with adsorbed oxygen to yield CO_2_ and H_2_O, as shown below:

5 $${\text{HCHO}} + 2{\text{O}}_{{({\text{ads}})}}^{ - } \to {\text{CO}}_{2} + {\text{H}}_{2} {\text{O}} + 2{\text{e}}^{ - }$$

At the end of the reaction process, the electrons held by the oxygen adsorbed will return to the conduction band of the material, resulting in a lower resistance of the sensor. The fabricated sensors performed better due to the high variation of resistance in air and formaldehyde vapor.

Compared to Bi_2_WO_6_ FMs, the response of Au@Bi_2_WO_6_ FMs to HCHO is greatly enhanced. The increased mechanism of the Au@Bi_2_WO_6_ FMs might be understood as follows: The work function of Au is (5.1 eV) greater than that of Bi_2_WO_6_ (2.6–2.7 eV), which leads to the transfer of electrons from Bi_2_WO_6_ to Au NPs. The contact is then established with a Schottky potential barrier. The potential barrier raises the resistance of the sensor. Meanwhile, the electron buildup on the surface of Au NPs causes adsorbed oxygen molecules to produce oxygen ions (${\text{O}}_{\text{2(ads)}}^{-}$ or ${\text{O}}_{\text{(ads)}}^{-}$) more quickly.

## Conclusion

We successfully created Bi_2_WO_6_ FMs and Au@Bi_2_WO_6_ FMs sensors and thoroughly investigated their sensing properties. It is found that the Au@Bi_2_WO_6_ FMs sensor's ideal working temperature (260 °C) is less than that of the Bi_2_WO_6_ FMs sensor (290 °C). The Bi_2_WO_6_ FMs sensor responded to 800 ppm formaldehyde with response of 230, but the Au@Bi_2_WO_6_ FMs sensor’s is 900, being about four times larger than the front. The response of the Au@Bi_2_WO_6_ FMs sensor to 100 ppm formaldehyde was also 2.5-fold as large as that of the untreated one. Therefore, Au@Bi_2_WO_6_ FMs sensor has a good response and selectivity to formaldehyde, making it an excellent candidate for formaldehyde detection.

## Data Availability

All data are fully available without restriction from the corresponding author on reasonable request.
